# Compliance with Washington State's requirement for residential carbon monoxide alarms

**DOI:** 10.1016/j.pmedr.2017.01.001

**Published:** 2017-01-12

**Authors:** Neil B. Hampson, James R. Holm

**Affiliations:** Center for Hyperbaric Medicine, Department of Medicine, Virginia Mason Medical Center, Seattle, WA, United States

**Keywords:** Carbon monoxide poisoning, Prevention, Alarms, Legislation

## Abstract

Carbon monoxide (CO) poisoning is responsible for significant morbidity and mortality in the US. In response, a majority of states have passed legislation in recent years requiring the installation of residential CO alarms. There is, however, no published information evaluating compliance with such laws. Employees of a Seattle medical center were surveyed in 2008 regarding home use of CO and smoke alarms. Washington State enacted legislation requiring residential CO alarms by all residences by January 1, 2013. The survey was repeated in mid-2016 to evaluate compliance. In 2016, a total of 354 employees completed the survey and their responses were compared to an equal number of 2008 survey respondents matched by home ownership and ZIP code. Residential CO alarm use rose from 37% to 78% (*p* < 0.0001). Among homeowners, 78% had alarms while 80% of renters had them. Homeowners with the highest compliance (96%) had purchased their homes since January 1, 2013 while those with the lowest compliance (73%) had purchased them earlier. A majority (79%) of renters without alarms reported the reason was that their landlord did not provide one, a violation of the law. Only one-half to two-thirds of all equipped homes had the required number of either CO or smoke alarms. Use of residential CO alarms increased significantly in this study population three years after law required them. Areas for further improvement include education of landlords, tenants, and longtime homeowners about the law, as well as public education regarding the number of CO and smoke alarms needed.

## Highlights

• Washington State has required residential carbon monoxide alarms since 2013.
• A survey on use of home CO alarms was conducted in 2008 and 2016.
• Residential CO alarm use increased from 41% to 78% overall.
• For the 20% of renters without alarms, most said their landlord didn't provide one.
• Use of home CO alarms increased significantly but further improvement is possible.

## 1 Introduction

Carbon monoxide (CO) poisoning accounts for hundreds of deaths and thousands of emergency department visits in the US annually (Centers for Disease Control and Prevention, 2007, Centers for Disease Control and Prevention, 2008, Hampson and Weaver, 2007, Hampson, 2016). It is generally believed that most accidental CO poisonings are preventable through use of public education, emission controls, warning labels on consumer products, and residential CO alarms. In an attempt to reduce the public's risk for poisoning, many states have passed legislation requiring the installation of residential CO alarms over the past several years.

As of January 1, 2016, thirty states had enacted statutes requiring installation of residential CO alarms in at least one category of domicile (National Conference of State Legislators). In response to a 2006 epidemic of storm-related CO poisoning in Washington (Gulati et al., 2009), that state's legislature passed a law mandating phased-in requirements for residential CO alarms (City of Seattle). Beginning January 1, 2011, state law required CO alarm installation in all newly constructed single-family homes and other residences. As of January 1, 2013, alarms were required in all existing residences. Certain single-family homes occupied prior to 2008 are exempted but will be required to have alarms when they are sold.

While it is the obvious intent of such legislation to achieve a high penetration of CO alarms into the homes of the targeted population, no information has been published to date examining the effectiveness of any state's legislation requiring residential CO alarm installation. In addition, a recent analysis of US CO-related mortality found no difference in the rates of decline in accidental deaths between states with and without laws requiring home residential alarms (Hampson, 2016). One potential explanation was that compliance with such laws might be poor or slow to occur.

A 2008 survey performed among employees of a Seattle medical center compiled responses from 574 households to a series of questions regarding home use of CO and smoke alarms (Hampson and Weaver, 2011). This study was conducted approximately four years before state alarm legislation was enacted and revealed that a minority of respondents had home CO alarms. In an attempt to evaluate compliance with laws requiring residential CO alarms, the identical survey was conducted in the same institution's employee population in 2016, three years after alarms became required by law (City of Seattle).

## 2 Methods

Virginia Mason Medical Center (VMMC) in Seattle is a private, nonprofit, multispecialty integrated medical center with 5041 employees at the time of this study in August 2016. Following approval by VMMC administration and the Institutional Review Board, the same 13-question survey on employee home use of CO and smoke alarms administered in 2008 was repeated. Full details regarding the survey and its administration can be found in the publication describing the 2008 results (Hampson and Weaver, 2011).

In brief, questions in the survey inquired about the presence of residential CO and smoke alarms, reasons for failure to have them, nature of home with regard to style, number of floors, owner vs. renter status, energy sources, and home ZIP code. One new question was added asking whether the respondent had moved into their current home prior to or after January 1, 2013. A notice on the institution's intranet home page solicited voluntary employee participation in the survey. Those wishing to participate were directed to a site where the questions were administered confidentially and anonymously through a commercial online survey company (www.surveymonkey.com). A total of 574 individuals participated in the 2008 survey and 365 in the 2016 survey.

Of the 365 responding in 2016, 9 surveys were incomplete and 2 listed home ZIP codes outside the State of Washington. After these 11 were excluded, the remaining 354 households, distributed among 96 ZIP codes, comprised the study population. The responses of 354 individuals from the larger 2008 respondent pool were used for comparison. These were selected randomly from among those with the same home ownership status and the same or nearby ZIP code as each of the 2016 respondents. To insure that the subgroup reflected the responses of the entire 2008 group, presence of smoke alarms and presence of CO alarms were compared between the total 2008 population of 574 and the subgroup of 354 selected as comparators for the 2016 survey population.

In addition to calculation of frequency of use of home alarms and the reasons for not having one, the data also allowed determination of whether homes having alarms met standards for number of alarms required. For carbon monoxide alarms, Washington State law requires placement of one outside each sleeping area (part of home where bedrooms are located) and one on each floor (City of Seattle). The minimum required for a one bedroom, one floor apartment would be one alarm. Each additional floor or sleeping area would add one alarm. Thus, the number of alarms required is one per floor, unless there is more than one sleeping area on a single floor. The National Fire Protection Agency guidelines require one smoke alarm inside each bedroom, one outside each sleeping area and one on each floor (National Fire Protection Agency). The minimum required for a one bedroom, one floor apartment would be two alarms. Each additional floor, sleeping area, or bedroom would add one alarm. Thus, the minimum required is the number of floors in the home plus one for a single bedroom.

To assess any role of socioeconomic status, median income of each respondent's ZIP code was compared with regard to variables such as home ownership and presence of a CO or smoke alarm (Cubit Inc).

Statistical significance was determined by two-tailed Fisher's Exact Test for binary outcomes between groups and unpaired Student's *t*-test for comparison of continuous variables.

## 3 Results

The subgroup of 2008 survey responders used as comparators with the 2016 survey results was not statistically different from the total 2008 survey population of 574 with regard to use of CO alarms (38.7% vs. 37.0%; *p* = 0.4518), or use of smoke alarms (98.4% vs. 98.9%; *p* = 0.3129). Table 1 lists demographics of the two populations compared in this study. There were no significant differences with regard to home type, number of floors, or presence of fuel-burning appliances.

Between 2008 and 2016, residential CO alarm use increased from 37% to 78% in the study population (*p* < 0.0001) (Table 2). Among homeowners, the 2016 rate of alarm use was 78%, while 80% of renters reported having CO alarms. Of those with alarms in 2016, the number of CO alarms per household was reported as one in 54%, two in 30%, three in 9%, and four or more in 7%.

Reasons given for not having a CO alarm are listed by home ownership status in Table 3. For homeowners without CO alarms, the most common reason given (43%) was “I haven't gotten around to it.” Fifteen respondents who gave as their reason for not having an alarm, “I am not at risk for carbon monoxide poisoning in my home.” Among these, 9 indicated that their home was all electric while 6 reported at least one fuel-burning appliance in their home.

Among homeowners who moved into their current home on or after January 1, 2013, the date CO alarms became mandatory in single-family homes at the time of sale, 50 of 52 (96%) had CO alarms. Of those living in owned homes since prior to that date, only 154 of 212 (73%) reported having an alarm.

CO alarm prevalence increased more among renters than homeowners, going from 12% in 2008 to 80% in 2016. Of those having alarms in 2016, the number of CO alarms per household was reported as one in 81%, two in 16%, three in 1%, and four or more in 1%. Of the 19 rental households reporting no CO alarm, 15 (79%) chose as the reason, “My landlord does not provide one.”

Smoke alarm use was nearly universal, similar in both 2008 and 2016, at 98–99% in aggregate and all subgroups (Table 2).

With regard to number of CO alarms in residences so equipped, 68% of homes had a sufficient number of alarms. For homes with smoke alarms, numbers reported were sufficient in 52%.

Mean household incomes were greater in residential ZIP codes of homeowners than renters ($77,585 ± 21,022 vs. $68,489 ± 14,924; *p* < 0.0001). Income did not differ between those who had CO alarms and those without ($74,479 ± 19,119 vs. $75,089 ± 22,966; *p* = 0.8027).

## 4 Discussion

Residential CO alarm use increased significantly in this Washington State population from 2008 to 2016, associated with full enactment of legislation requiring them on January 1, 2013. Use doubled in the aggregate population, rising from 37% to 78%. The largest gains were seen among renters, among whom alarm use increased over six-fold, from 12% to 80%. Use among homeowners also increased, but to a lesser degree. The group with the lowest 2016 rate of alarm use (73%) was homeowners who had lived in their homes over three years.

A number of observations can be made regarding these results. First, the law requiring residential alarms is having an effect. In addition to the dramatic increase described among renters, homeowners who moved into (and presumably purchased) homes after the date requiring them as a contingency of sale had a compliance rate of 96%, similar to smoke alarm usage and essentially achieving the goal of uniform installation.

As is typical of many state alarm laws, the Washington law required the devices initially in new construction and rental units, with owners of pre-existing single-family homes receiving more latitude. While alarms have been required in most categories of residence since January 1, 2013, enforcement in owned single-family homes only occurs when the home is sold. This is evident when comparing rates of use between those occupying single-family homes prior to and after that date (73% vs. 96%).

Widespread lack of knowledge about the alarm law did not seem to be a significant issue, as only 10% selected as their reason for noncompliance as, “My community's building codes do not require them.” However, there may be a role for more education among some landlords and renters. Alarms were required in all rental units for more than three years at the time of the survey, yet one renter in five reported not having one. Of these, the vast majority noted that their landlord had not provided one. This was true whether the rental was recent or longstanding.

The group of longstanding homeowners without alarms most commonly gave the reason, “I haven't gotten around to it.” This implies that they are aware alarms exist and possibly know of the legal requirement as well, but have not been motivated to proceed. Nonetheless, their 73% usage rate represents a 40% increase since 2008. As this was not the result of a home sale requirement, it presumably represents the impact of public education.

When socioeconomic status is examined, average median income by ZIP codes of homeowners was higher than that of renters. One might expect that absence of a CO alarm would correlate with lower income, but this was not the case. Since the law requires landlords to provide alarms for renters, it is not a discretionary purchase affected by income. Also, despite the higher income of the homeowner group, those who purchased their homes prior to the 2013 deadline have not been required to install alarms until the time of home sale.

When one examines the number of each alarm type in equipped homes, only 68% of homes had a sufficient number of CO alarms and only 52% had a sufficient number of smoke alarms. These numbers may be overestimates of true compliance, since they were calculated assuming only one bedroom and sleeping area per home. An insufficient number of alarms is likely the result of consumer confusion because the formulas used to calculate the required number of each type of alarm use different permutations of the number of home floors, bedrooms, and sleeping areas.

While legislation mandating residential CO alarms in Washington State is having an effect, opportunities for improvement remain. Tenants should be educated that it is their right to ask a landlord install a CO alarm in their rental unit. Similar information should be promulgated to landlord associations. The law's requirement that every home sold must have a CO alarm appears to be accomplishing its purpose, with near uniform application in that subgroup. Longer-term homeowners have increased their usage since 2008 but continue to lag behind the other groups. Since they will be required to have a CO alarm when they eventually sell their homes, installation at some point is necessary. If done now, they could benefit from the protection provided by the device in the meantime.

The public should receive continual education regarding alarm maintenance and routine testing. Testing will teach the sound their device makes when in alarm mode. Education should also include recommendations for necessary action when the alarm sounds.

It is not surprising that only two-thirds of homes having either device are equipped with a sufficient number. With different requirements for CO and smoke alarms, it is easy to become confused. A potential solution is promotion of combination smoke and CO alarms. Following the smoke alarm guidelines and installing combination alarms would satisfy the CO alarm requirements, as well. This has not been practical in the past because CO alarms required replacement every five or seven years, depending on the model, while smoke alarms required replacement every ten years. Now, several models of combination detectors effective for ten years and containing a sealed ten-year battery are being marketed (First Alert, n.d, Kidde, United Technologies, n.d, Nest, n.d).

One potential limitation to this study is the possibility that survey respondents inaccurately reported the number of alarms in their homes, thereby influencing the frequencies that an adequate number of either type were present. Secondly, the physical presence of an alarm does not guarantee that it is operational. If proper maintenance and scheduled alarm replacement are not performed, the batteries or device itself could be expired.

Finally, this study may help explain the observation that the rates of decline in mortality from accidental CO poisoning are similar in states with and without laws requiring residential CO alarms (Hampson, 2016). While compliance with the law in Washington is impressive, it is not universal three years after implementation. In addition, an increase in alarm installation has occurred even among that segment of the population in which their presence has not yet been mandated by home sale. If this is occurring across the country as a result of public education, the mortality differences between states with and without residential CO alarm laws may be minimized.

## Figures and Tables

**Table 1:** Study population characteristics 2008 vs. 2016.

	2008	2016
Medical center employees	4909	5041
Survey respondents studied (% of employees)	354 (7%)	354 (7%)
Home type		
Single-family	68%	70%
Apartment type	21%	22%
Duplex/townhome	8%	7%
Modular/manufactured	2%	1%
Number of Floors in Home		
1	31%	36%
2	49%	44%
3	16%	16%
4	3%	2%
All electric appliances (no fuel-burning)	36%	36%

**Table 2:** Prevalence of CO and smoke alarm use in 2008 and 2016 surveys by home ownership status.

	2008 Survey	2016 Survey	
Have residential carbon monoxide alarm	37% overall	78% overall	*p* < 0.0001
Homeowners	46%	78%	*p* < 0.0001
Moved in before January 1, 2013		73%	
Moved in January 1, 2013 or later		96%	
Renters	12%	80%	*p* < 0.0001
Moved in before January 1, 2013		77%	
Moved in January 1, 2013 or later		81%	
Have residential smoke alarm	99% overall	98% overall	
Homeowners	99%	98%	
Renters	99%	99%	

**Table 3:** Reasons given by 2016 survey respondents for not having a home CO alarm. Percentages sum to > 100% because some respondents gave more than one reason.

Why don't you have a CO alarm?	Owners (*n* = 58)	Renters (*n* = 19)
I have not gotten around to it.	43%	21%
I′m not at risk for CO poisoning in my home.	21%	16%
They are not required in my community.	10%	11%
They are too expensive.	9%	5%
I did not know they existed.	7%	5%
I have a smoke alarm.	5%	5%
I don't know where to buy one.	3%	11%
My landlord does not provide one.	N/A	79%

## Appendix A Supplementary data

Survey Questions Image 1
