# Supplementary material for: Compliance with Washington State's requirement for residential carbon monoxide alarms
Source: Prev Med Rep. 2017 Jan 12;5:232–5. doi: 10.1016/j.pmedr.2017.01.001 (PMC5247563; doi:10.1016/j.pmedr.2017.01.001)
Supplement: Supplementary file 1 — Survey Questions [file mmc1.pdf]

* 1. Do you have a SMOKE detector or alarm in your home?

- ◯ Yes
- ◯ No

* 2. How many SMOKE detectors or alarms do you have in your home?

- [ ] 1
- [ ] 2
- [ ] 3
- [ ] 4 or more

* 3. How are your SMOKE detectors or alarms powered?

- [ ] Household AC current (plugged into wall outlet or wired in)
- [ ] Batteries
- [ ] Household AC current with battery back-up
- [ ] Unknown

4. Why don't you have a SMOKE detector or alarm in your home? (mark as many answers as apply)

- [ ] I do not know where to buy one.
- [ ] I did not know they existed.
- [ ] They are too expensive.
- [ ] My landlord does not provide one.
- [ ] My community's building codes do not require them.
- [ ] I have a carbon monoxide detector or alarm and do not need one for smoke, too.
- [ ] I just have not gotten around to it.
- [ ] I am not at risk for fire in my home.

Other (please specify)

* 5. Do you have a CARBON MONOXIDE detector or alarm in your home?

- ◯ Yes
- ◯ No

* 6. How many CARBON MONOXIDE detectors or alarms do you have in your home?

- ◯ 1
- ◯ 2
- ◯ 3
- ◯ 4 or more

* 7. How are your CARBON MONOXIDE detectors or alarms powered?

- ◯ Household AC current (plugged into wall outlet or wired in)
- ◯ Batteries
- ◯ Household AC current with battery back-up
- ◯ Unknown

8. Why don't you have a CARBON MONOXIDE detector or alarm in your home? (mark as many answers as apply)

- [ ] I do not know where to buy one.
- [ ] I did not know they existed.
- [ ] They are too expensive.
- [ ] My landlord does not provide one.
- [ ] My community's building codes do not require them.
- [ ] I have a smoke detector or alarm and do not need one for carbon monoxide, too.
- [ ] I just have not gotten around to it.
- [ ] I am not at risk for carbon monoxide exposure in my home.

Other (please specify)

* 9. What best describes your home?

- ◯ Single family house
- ◯ Apartment/apartment-type
- ◯ Duplex/townhouse
- ◯ Modular/manufactured

10. When did you move into your current home?

- ◯ January 1, 2013 to present
- ◯ Before January 1, 2013

* 11. Do you rent or own your home?

- ◯ Rent
- ◯ Own

* 12. What is your home ZIP code?

Enter 5 digit ZIP code in box. [ ]

* 13. How many floors are there within your home?

- ◯ 1
- ◯ 2
- ◯ 3
- ◯ 4 or more

14. With regard to your home heating, stove and water heater, do ANY of them use natural gas, oil or propane?

- ◯ Yes, at least one uses gas, oil or propane
- ◯ No, all are electric

Thank you for your participation.
